# Polygyny and intimate partner violence in sub-Saharan Africa: Evidence from 16 cross-sectional demographic and health surveys

**DOI:** 10.1016/j.ssmph.2021.100729

**Published:** 2021-01-12

**Authors:** Bright Opoku Ahinkorah

**Affiliations:** School of Public Health, Faculty of Health, University of Technology Sydney, Sydney, Australia

**Keywords:** Polygyny, Intimate partner violence, Sub-saharan Africa, Global health

## Abstract

In sub-Saharan Africa, where intimate partner violence has been found to be predominant, several scholars have made efforts to understand its predictors. Socio-culturally, polygyny has been considered as a key determinant of intimate partner violence. This study aimed to examine the association between polygyny and intimate partner violence in 16 sub-Saharan African countries. Binary logistic regression models were used in testing the association and the results were presented as crude and adjusted odds ratios at 95% confidence interval. The proportion of women in polygamous marriages in the 16 countries was 20.2%, ranging from as high as 40% in Chad to as low as 1.6% in South Africa. The prevalence of IPV was 30.7% in the 16 countries, ranging from as high as 44% in Uganda to as low as 12.7% in South Africa. The odds of IPV were higher among women in polygamous marriages in Angola, Burundi, Ethiopia, Uganda, Malawi, Mozambique, Zambia and Zimbabwe but was lower among women in polygamous marriages in Cameroon [COR = 0.54, 95% CI = 0.44–0.66] and Nigeria [COR = 0.61, 95% CI = 0.55–0.67], and this persisted after controlling for level of education, place of residence, wealth quintile, media exposure, and justification of violence. This study has found a significant association between polygyny and intimate partner violence. The practice of intimate partner violence in sub-Saharan Africa is fused into the socio-cultural norms and religious traditions of most countries in the sub-Saharan African region. The findings imply that family structures expose women to intimate partner violence. Therefore, global efforts in dealing with intimate partner violence through the Sustainable Development Goals should be done with attention on the socio-cultural norms and traditions around marriage and family structures.

## Highlights

• Global estimates on intimate partner violence show that one in three women has experienced intimate partner violence at some point in her life.
• Sub-Saharan Africa is the region with the highest prevalence of intimate partner violence globally (36%).
• Socio-culturally, polygyny has been considered as a key determinant of intimate partner violence.
• Global efforts in dealing with intimate partner violence should be done with focus on the socio-cultural norms and traditions around marriage and family structures.

## Background

Gender-based violence (GBV) is a notable element of human rights violation, and intimate partner violence (IPV) has been regarded as the most significant component of GBV (Ahinkorah, Dickson, & Seidu, 2018; Devries et al., 2013). IPV has been described as an influential and multi-faceted problem in society that is linked with numerous social and health consequences (Organisation, 2013; Rahman, Nakamura, Seino, & Kizuki, 2013). It includes physical, sexual, and emotional abuse and controlling behaviours by an intimate partner (World Health, 2012). Some of the wide range of negative consequences of IPV for women include loss of pregnancy, through stillbirths and miscarriages, and contraction of sexually transmitted infections (Durevall & Lindskog, 2015). Women who suffer from IPV are also more likely to report high levels of depression, post-traumatic stress disorder, psychological distress, as well as suicidal thoughts (Mason & Lodrick, 2013; Nguyen et al., 2019; Ogunwale & Oshiname, 2017).

Global estimates on IPV show that one in three women has experienced IPV at some point in her life, although there are variations in these estimates between countries (Abramsky et al., 2011; Jewkes et al., 2017). Sub-Saharan Africa (SSA) is the region with the highest prevalence of IPV globally (Devries et al., 2013), with an overall prevalence of 36% considered to be higher than the global average of 30% (García-Moreno et al., 2013). Majority of women in Africa have experienced lifetime IPV (45.6%) and sexual assault (11.9%) than women anywhere in the world (García-Moreno et al., 2013).

As part of global efforts to deal with violence against women, the United Nations Sustainable Development Goals (SDGs) aim to eradicate all forms of violence against women and ensure that all countries are free from IPV by 2030, taken into consideration the deep rooted practices and effects of GBV against women (United Nations, 2015). In line with this, target 5.2 of the SDGs focuses on ensuring the elimination of all forms of violence against all women and girls in public and private spheres, including trafficking and sexual and other types of exploitation by 2030 and target 16.1 is also geared towards reducing significantly all forms of violence and related death rates everywhere by 2030 (United Nations, 2015). To achieve this, it is important that all stakeholders globally improve and work towards decreasing the prevalence of GBV, including IPV (Garcia-Moreno & Amin, 2016). Therefore, understanding the prevalence and causes of IPV can be an essential first step by government and non-government organisations in helping to achieve SDG 5.2 and 16.1 through appropriate and effective policy response.

In SSA, where IPV has been found to be predominant, several scholars have made efforts to understand the predictors of IPV, with some identifying socio-demographic factors such as age at first marriage, spousal age difference, education, wealth index, place of residence, among other factors as predictors (Ahinkorah et al., 2018; Akamike, Uneke, Uro-Chukwu, Okedo-Alex, & Chukwu, 2019; Dim, 2020; Izugbara, Obiyan, Degfie, & Bhatti, 2020; McCloskey, Boonzaier, Steinbrenner, & Hunter, 2016; Yaya, Hudani, Buh, & Bishwajit, 2019). Other studies have established an association between women's autonomy or household decision-making capacity and IPV (Ahinkorah et al., 2018; Tenkorang, 2018; Zegenhagen, Ranganathan, & Buller, 2019).

Socio-culturally, polygyny has been considered as a key determinant of IPV and recent studies on IPV in SSA have tried to understand the association between polygyny and IPV (Heath, Hidrobo, & Roy, 2020; N. Jansen & Agadjanian, 2020; N. A. Jansen & Agadjarian, 2016). Polygyny has been defined as the practice of one man being married to multiple wives at the same time (Smith-Greenaway & Trinitapoli, 2014) and this practice has been found to be very common in SSA (Amo-Adjei & Tuoyire, 2016; Uthman, Lawoko, & Moradi, 2010). Scholars that have examined the association between polygyny and IPV have often found that the odds of IPV is higher among women with co-wives compared to those in monogamous marriages (Behrman, 2019; Ebrahim & Atteraya, 2020; Heath et al., 2020; N. Jansen & Agadjanian, 2020; N. A. Jansen & Agadjarian, 2016). Unfortunately, these studies have been conducted only in specific countries in SSA such as Ethiopia (Ebrahim & Atteraya, 2020), Mozambique (N. Jansen & Agadjanian, 2020; N. A. Jansen & Agadjarian, 2016), Mali (Heath et al., 2020) and Nigeria (Behrman, 2019).

Since IPV is a major social and health issue in SSA, a more expanded study that uses nationally-representative data to examine the association between polygyny and IPV and subjects to test the already established association between the two phenomena in countries within SSA is worthwhile. In line with this, this study aimed to examine the association between polygyny and IPV in 16 sub-Saharan African countries using data from the demographic and health surveys (DHS). Findings from this study will not only provide evidence of how polygyny plays a role in IPV in each of these countries, but it will also be a benchmark for government and non-governmental organisations in the 16 sub-Saharan African countries to initiate policies and programs that will help end IPV.

## Methods

### Study design

This was a cross-sectional study carried out using pooled DHS data of 16 countries in SSA. DHS is a nationally representative survey that employs multistage sampling design, to gather data across low- and middle-income countries every five years. Details on the sampling methodology and data collection used by the DHS are published elsewhere (Corsi, Neuman, Finlay, & Subramanian, 2012). In this study, countries were included if they had information on the DHS domestic violence modules and had openly available datasets obtained between 2015 and 2018. One reason for limiting the analyses to data published between 2015 and 2019 was to examine current data in line with the SDGs which were published in 2015 and have specific goals (SDG-5.2 and SDG-16-1) aimed at dealing with intimate partner violence (United Nations, 2015). Limiting the analysis to recent DHS also reflects the overarching need for current, up-to-date evidence to inform policy debates on dealing with intimate partner violence in SSA. Although 17 countries had data published between 2015 and 2018, only 16 of them had data on domestic violence. The excluded country was Guinea. In all, 56,804 married women were included in this study. The countries included in this study are shown in Table 1. The manuscript was prepared in line with the Strengthening Reporting of Observational studies in Epidemiology (STROBE) reporting guidelines (Von Elm et al., 2014).

**Table 1:** Sample distribution by country.

Survey Countries	Survey Year	Weighted Sample	Percentage
Central Africa			
Angola	2016	1653	2.91
Cameroon	2018	3008	5.3
Chad	2015	3182	5.60
**West Africa**			
Benin	2018	3231	5.69
Mali	2018	3366	5.93
Nigeria	2018	8156	14.36
**East Africa**			
Burundi	2017	4387	7.72
Ethiopia	2016	4097	7.21
Rwanda	2015	990	1.74
Tanzania	2016	4529	7.97
Uganda	2016	2937	5.17
**Southern Africa**			
Malawi	2016	4190	7.38
Mozambique	2015	1439	2.53
South Africa	2016	1250	2.20
Zambia	2018	5721	10.07
Zimbabwe	2015	4669	8.22

### Study variables

#### Outcome variable

The outcome variable for the study was IPV. Three key variables (sexual violence, emotional violence and physical violence) were used to generate this variable. These were derived from the optional domestic violence module, where questions are based on a modified version of the conflict tactics scale (Kishor, 2005; Straus, 1979). Questions asked are related to the experience of physical, emotional, or sexual violence. In this study, the focus was on the experience of physical, emotional or sexual violence in the last 12 months. Questions on physical violence used in this study include whether respondent's last partner ever: pushed, shook or threw something at her; slapped her; punched her with his fist or something harmful; kicked or dragged her; strangled or burnt her; threatened her with a knife, gun or other weapons; and twisted her arm or pulled her hair. On emotional violence, a respondent was asked if her last partner ever: humiliated her; threatened to harm her; and insulted or made her feel bad. There are three standard questions on sexual violence: whether the partner ever physically forced the respondent into unwanted sex; whether the partner ever forced her into other unwanted sexual acts; and whether the respondent has been physically forced to perform sexual acts she didn't want to. For each of these questions, the responses were ‘never’ ‘often’ ‘sometimes’ and ‘yes, but not in the last 12 months’. For physical, emotional and sexual violence, a dichotomous variable was created to represent whether a respondent had experienced any of these forms of violence in the past 12 months by coding never and yes, but not in the last 12 months together as ‘No’ and often and sometimes, coded together as ‘Yes’. Finally, a third variable, known as experienced IPV in the last 12 months was created to represent whether a respondent had reported experiencing either physical, emotional and/or sexual violence in the past 12 months. The analysis was limited to experience of IPV in the past 12 months to reduce the bias lifetime experience of IPV could bring since the focus of the study was to look at polygyny within currently married women and that past year experience of IPV may have occurred within the current union.

#### Key explanatory variable

The key explanatory variable in this study was polygyny. This variable has been defined in the DHS as the number of other wives that the partner of currently married women (women who are either legally or formally married or who are living in a consensual union) has. Reference to the use of this variable has been published elsewhere (Anjorin et al., 2020; Smith-Greenaway & Trinitapoli, 2014). However, in this study, only currently married women were considered in order to understand the link between current marital structure and IPV. Following the use of this variable in previous studies, women who indicated that their partners had no other wives were considered as being in monogamous marriages whiles those who indicated that their partners had 1 or more other wives were considered as those in polygamous marriages. Hence, a dichotomous outcome variable was derived from the variable polygyny and was coded as 0 = monogamy and 1 = polygamy.

### Covariates

Five important socio-demographic covariates (level of education, place of residence, wealth quintile, media exposure, and justification of violence) were included in the analysis to adjust for the modelling. The selection of these variables was based on their availability in the datasets and their statistically significant associations with IPV in previous studies (Ahinkorah et al., 2018; Izugbara et al., 2020; McCloskey et al., 2016; Muluneh, Stulz, Francis, & Agho, 2020). In the DHS, level of education was coded as no education, primary, secondary and higher. However, for the purpose of the analysis in this study, the variable was recoded as no education, primary and secondary/higher. Place of residence was coded as “rural” and “urban” in the DHS and this was adopted in this study. Wealth quintile in the DHS was assessed as an index of household assets and utilities using Principal Component Analysis (PCA) and categorised as “poorest” “poorer”, “middle”, “richer” and “richest”. In this study, the original categorization of wealth quintile as used in the DHS was adopted. Media exposure was created from three variables on the frequency of watching television, listening to radio, or reading newspaper/magazine. The respondents were assigned 0 for “not at all”, 1 for “less than once a week” and 2 for “at least once a week”. These responses were further re-categorised into ‘No’ (not at all) and “Yes” (less than once a week and at least once a week). After this, a dichotomous variable was created from a composite of exposure to the three media sources and defined as “No” and “Yes”. Finally, justification of violence was a variable that was derived from a question in the DHS that asked women if a husband is justified of wife beating for the following reasons: (i) burning food (ii) arguing with him (iii) going out without telling him (iv) neglecting the children, and (v) refusing to have sexual intercourse with him (Hamilton, 2012). A binary variable was created from these five reasons to reflect the attitudes towards wife beating. Justification of violence was therefore coded as ‘no’ if the women did not agree with any of the five reasons and ‘yes’ if she agreed to at least one of these reasons for wife beating.

### Data analyses

**Fig. 1:**
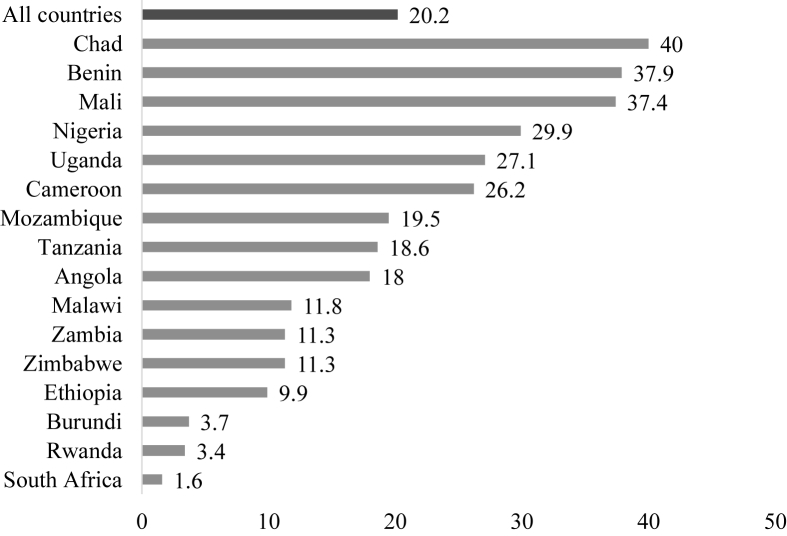
Proportion of women in polygamous marriages in sub-Saharan Africa.

**Fig. 2:**
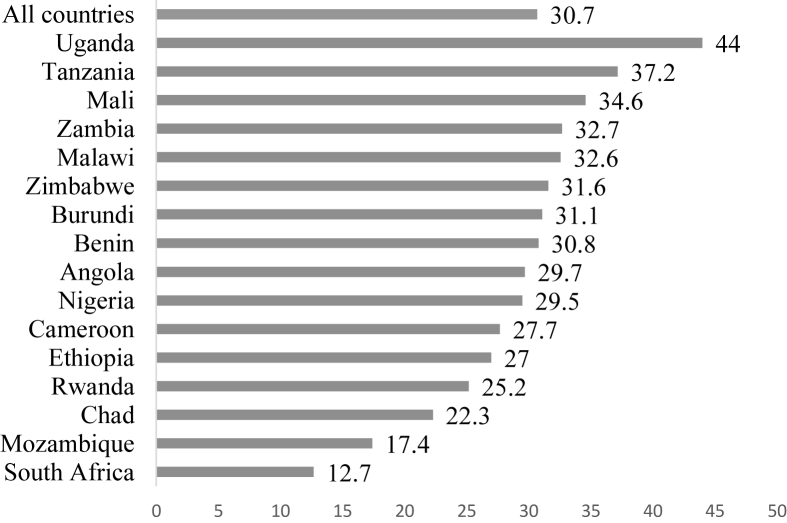
Prevalence of IPV in sub-Saharan Africa by country.

**Fig. 3:**
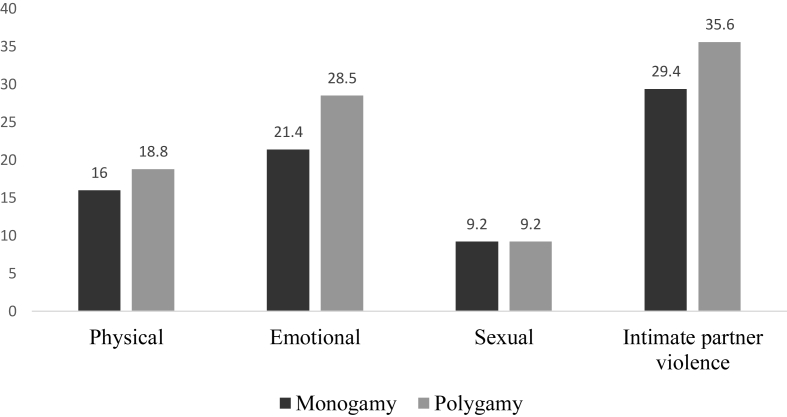
Polygyny and past-year intimate partner violence.

Using Stata Version 14.0, data analyses in this study first begun with the calculation of the proportion of women who had experienced IPV in the last 12 months and those who were in polygamous marriages using percentages and presented using bar charts (see Fig. 1, Fig. 2). Next, the results of the distribution of physical, emotional, sexual, and intimate partner violence across the two categories of polygyny were also presented using bar charts (see Fig. 3). This was followed by the use of Pearson's chi-square test to examine the relationship between polygyny and physical, emotional, sexual, and intimate partner violence in each of the 16 countries in SSA. Finally, the effect of polygyny on IPV in each of the 16 countries in SSA was assessed using both bivariate and multivariable binary logistic regression models. The results of the regression analyses were presented as crude odds ratios (COR) and adjusted odds ratios (AOR), at 95% confidence intervals (CIs). The women's sample weights for the domestic violence module (d005/1,000,000) were applied to obtain unbiased estimates, according to the DHS guidelines and the survey command (SVY) in Stata was used to adjust for the complex sampling structure of the data in the regression analyses.

## Results

### Prevalence of polygyny in sub-Saharan Africa

The proportion of women in polygamous marriages in the 16 countries was 20.2%, ranging from as high as 40% in Chad to as low as 1.6% in South Africa (Fig. 1).

### Prevalence of intimate partner violence in sub-Saharan Africa

The prevalence of IPV in the 16 countries was 30.7%, ranging from as high as 44% in Uganda to as low as 12.7% in South Africa (Fig. 2).

### Distribution of polygyny across physical, emotional, sexual and intimate partner violence

Compared to women in marriages with no co-wives (16%), those in polygamous marriages had the highest prevalence of physical violence (18.8%). Past year experience of emotional violence was higher among women whose husbands had additional wives (28.5%), compared to those in monogamous marriages (21.4%). Compared to women in monogamous marriages (29.4%) those in polygamous marriages (35.6%) had the highest prevalence of IPV in the last 12 months. However, past year experience of sexual violence was evenly distributed among women in monogamous and polygamous marriages (9.2% for each category of polygyny) (see Fig. 3).

Table 2 shows the distribution of polygyny across past year experience of physical, emotional, sexual and intimate partner violence by countries. In general, past year experience of any IPV was higher among women in marriages with one or more co-wives compared to monogamous marriages in all the 16 countries, except Cameroon, where the reverse was true. Moreover, the association between polygyny and IPV was statistically significant at 95% CI in 11 (Angola, Benin, Mali, Burundi, Ethiopia, Tanzania, Uganda, Malawi, South Africa, Zambia and Zimbabwe) out of the 16 countries in SSA considered in this study.

**Table 2:** Polygyny and past-year intimate partner violence by countries.

Countries	Physical violence		p-values	Emotional violence		p-values	Sexual violence		p-values	Any IPV		p-values
	monogamy	polygamy		monogamy	polygamy		monogamy	polygamy		monogamy	polygamy	
**Central Africa**												
Angola	19.9	30.4	0.008	18.1	27.6	0.015	4.5	7.2	0.181	26.7	42.6	0.001
Cameroon	16.4	16.6	0.947	20.3	18.0	0.424	5.8	2.3	0.003	28.4	25.9	0.520
Chad	13.4	16.1	0.135	14.0	17.2	0.073	7.7	5.9	0.215	21.4	23.7	0.310
**West Africa**												
Benin	8.5	10.8	0.048	25.3	32.0	0.001	5.5	4.7	0.387	28.1	35.2	0.001
Mali	17.2	20.0	0.119	26.1	32.7	0.002	6.7	10.3	0.011	31.8	39.3	0.002
Nigeria	11.1	11.4	0.814	26.0	28.7	0.079	4.1	5.2	0.124	28.6	31.6	0.076
**East Africa**												
Burundi	15.7	34.5	<0.001	14.1	33.9	<0.001	18.2	35.6	<0.001	30.1	56.8	<0.001
Ethiopia	15.8	23.1	0.023	18.9	30.0	0.001	8.3	10.6	0.325	26.0	36.3	0.011
Rwanda	16.4	24.4	0.268	15.5	36.6	0.003	7.6	13.3	0.242	24.8	36.6	0.148
Tanzania	24.2	31.6	0.002	26.4	34.2	0.001	9.3	9.4	0.950	35.9	43.1	0.017
Uganda	22.4	28.9	0.002	31.6	36.1	0.051	17.3	18.8	0.408	42.2	48.6	0.010
**Southern Africa**												
Malawi	14.9	19.1	0.087	21.7	29.9	0.001	14.7	20.2	0.018	31.5	40.7	0.001
Mozambique	13.2	10.0	0.297	10.5	13.6	0.261	2.3	1.5	0.546	17.3	18.2	0.799
South Africa	5.6	17.6	0.021	8.7	27.8	0.004	2.3	2.3	0.606	12.4	31.0	0.014
Zambia	19.7	30.1	<0.001	21.2	30.6	<0.001	10.1	18.2	<0.001	31.4	43.3	<0.001
Zimbabwe	15.0	17.3	0.265	23.6	33.7	<0.001	8.6	13.4	0.013	30.3	41.9	<0.001

Note: Pearson chi-square test was used to obtain p-values.

### **Association between** polygyny and intimate partner violence in sub-Saharan Africa

To examine the association between polygyny and IPV, two models were fitted and the results have been presented in Table 3. Model I was a crude model with no covariates, while Model II adjusted for the covariates. In Model I, a statistically significant effect of polygyny on IPV was found. However, whereas the likelihood of IPV was higher among women in polygamous marriages in Angola, Burundi, Ethiopia, Uganda, Malawi, Mozambique, Zambia and Zimbabwe, the odds of IPV was lower among women in polygamous marriages in Cameroon [COR = 0.54, 95% CI = 0.44–0.66] and Nigeria [COR = 0.61, 95% CI = 0.55–0.67].

**Table 3:** Bivariate and multivariable models showing the effect of polygyny on IPV in selected sub-Saharan Africa countries.

Countries	Model I	Model II
	COR [95% CI]	AOR [95% CI]
Central Africa		
Angola	2.02*** [1.56–2.63]	1.91*** [1.45–2.50]
Cameroon	0.54*** [0.44–0.66]	0.58*** [0.47–0.72]
Chad	0.92 [0.78–1.09]	0.96 [0.81–1.14]
**West Africa**		
Benin	0.90 [0.79–1.03]	0.87 [0.75–1.00]
Mali	0.97 [0.85–1.12]	0.91 [0.79–1.05]
Nigeria	0.61*** [0.55–0.67]	0.62*** [0.56–0.69]
**East Africa**		
Burundi	2.34*** [1.79–3.07]	2.31*** [1.75–3.05]
Ethiopia	1.24* [1.03–1.49]	1.20 [0.99–1.44]
Rwanda	1.68 [0.97–2.90]	1.57 [0.90–2.73]
Tanzania	0.89 [0.77–1.03]	0.84* [0.72–0.98]
Uganda	1.32***[1.16–1.51]	1.21** [1.05–1.38]
**Southern Africa**		
Malawi	1.30** [1.12–1.52]	1.27** [1.09–1.49]
Mozambique	1.44* [1.01–2.06]	1.59* [1.11–2.29]
South Africa	2.14 [0.93–4.90]	1.52 [0.64–3.62]
Zambia	1.53*** [1.31–1.78]	1.45*** [1.24–1.70]
Zimbabwe	1.38**[1.14–1.68]	1.31** [1.07–1.60]

Model 1: unadjusted model examining the independent association of polygyny and intimate partner violence; Model 2: adjusted for socio-demographic factors (educational level, residence, wealth index, media exposure, and justification of violence); AOR is the adjusted odds ratio, UOR is the unadjusted odds ratio, ref is the reference; Exponentiated coefficients; 95% confidence intervals in brackets.

*p < 0.05, **p < 0.01, ***p < 0.001.

After controlling for the covariates, the magnitude and direction of association persisted, except in Ethiopia, where no statistically significant association was found between polygyny and IPV, showing a strong and robust association between polygyny and IPV. Countries with higher odds of IPV among women in polygamous marriages in the adjusted model were as follows: Angola [AOR = 1.91, 95% CI = 1.45–2.50], Burundi [AOR = 2.31, 95% CI = 1.75–3.05], Uganda [AOR = 1.21, 95% CI = 1.05–1.38], Malawi [AOR = 1.27, 95% CI = 1.09–1.48], Mozambique [AOR = 1.59, 95% CI = 1.11–2.29], Zambia [AOR = 1.45, 95% CI = 1.24–1.70] and Zimbabwe [AOR = 1.31, 95% CI = 1.07–1.60]. The odds of IPV was lower among women in polygamous marriages in Cameroon [AOR = 0.58, 95% CI = 0.47–0.72], Nigeria [AOR = 0.62, 95% CI = 0.56–0.69] and Tanzania [AOR = 0.84, 95% CI = 0.72–0.98] (see Model II of Table 3).

## Discussion

Global estimates on IPV show that more than 30% of women have experienced IPV at some point in their lives, although there are variations in these estimates between countries (Abramsky et al., 2011; Jewkes et al., 2017). In this study, the association between polygyny and IPV was examined. It was found that in the 16 countries studied, the prevalence of IPV was 30.7%, with the highest and lowest prevalence in Uganda and South Africa, respectively. The overall prevalence of 30.7% is similar but a bit lower than the 36% found in the study of García-Moreno et al. (2013). The difference in the prevalence could be attributed to the differences in study periods, number of countries involved in the study and sample. The high prevalence of IPV in Uganda (44%) confirms the findings of studies on IPV that were conducted in Uganda (Black et al., 2019; Gubi, Nansubuga, & Wandera, 2020; Prabhu et al., 2011). However, in these studies, the prevalence were higher than what was found in the current because the previous studies focused on lifetime experience of IPV, contrary to the past year experience of IPV in the current study. Polygyny was found to be 20.2% in the 16 countries considered in this study with high prevalence among women in West and Central African countries and this has been evidenced in a previous study (Fenske, 2015).

In this study, polygyny was found to be associated with IPV, with women in polygamous marriages having a higher prevalence of IPV, compared to those in marriages with no co-wives. Relatedly, the odds of past year experience of IPV was higher among women in polygamous marriages in seven of the 16 countries studied except Cameroon and Nigeria. Findings on the association between polygyny and IPV corroborates the findings of previous studies that were conducted in Ethiopia (Ebrahim & Atteraya, 2020), Mozambique (N. Jansen & Agadjanian, 2020; N. A. Jansen & Agadjarian, 2016), Mali (Heath et al., 2020) and Nigeria (Behrman, 2019), where the authors concluded that the likelihood of IPV is higher among women in polygamous marriages compared to those in monogamous marriages. One of the possible reasons for the finding is that in polygamous households, the interaction between household members is generally poorer due to competition over resources among senior wives and junior wives (Heath et al., 2020). In SSA, Bove and Valeggia (2009) found less spousal communication and weaker emotional ties in polygamous marriages, compared to monogamous marriages and this can induce IPV. It has also been established that IPV is more likely to occur in polygamous marriages due to low cooperation between spouses attributed to competition amongst co-wives and increased problems with coordination, information, and monitoring (Baland & Ziparo, 2017; Rossi, 2019). The finding that women in monogamous marriages are more likely to experience IPV in Nigeria contradicts the findings of a previous study conducted in Nigeria by Ashimi and Amole (2015). The possible reason for the difference in findings could be the differences in study sample. This is because, while the focus of the current study was on married women, that of the study by Ashimi and Amole (2015) was on pregnant women. Notwithstanding, the relatively high prevalence of IPV among women in polygamous marriages in most of the countries studied in the current study provides evidence of multi-faceted socio-cultural perspective in dealing with IPV.

## Strengths and limitations

The strength of this study is the use of relatively large datasets that are nationally representative to examine the association between polygyny and IPV. Again, the statistical analyses performed using the large sample in this study supports the accuracy of the findings. Despite these strengths, it is worth acknowledging the limitations inherent in this study. First, the surveys used in this study were based on cross-sectional data, and hence, causal interpretations of the findings on the association between polygyny and IPV cannot be established. Second, past year experience of IPV was also self -reported, and as a result, there is the possibility of under-and over-reporting of data.

## Conclusion

This study has found a significant association between polygyny and IPV. The practice of IPV in SSA is fused into the socio-cultural norms and religious traditions of most countries in the sub-Saharan African region. The findings imply that such family structures expose women to IPV. Thus, policies and programmes aimed at dealing with IPV should pay particular attention to women in polygamous marriages. The findings also indicate the social complexity of polygamous marriages and the resulting vulnerabilities it poses on women. Therefore, global efforts in dealing with IPV through the SDGs should be done with attention to the socio-cultural norms and traditions around marriage and family structures.

## Ethical statement

Ethical permissions were not required for this study since DHS datasets which is publicly available was used. Institutions that commissioned, funded, or managed the surveys were responsible for ethical procedures. ICF international as well as an Institutional Review Board (IRB) in each respective country approved all the DHS surveys in line with the U.S. Department of Health and Human Services regulations for the protection of human subjects. Data is available on https://dhsprogram.com/data/available-datasets.cfm.

## Financial disclosure

There is no funding for this study.

## CRediT authorship contribution statement

**Bright Opoku Ahinkorah:** Conceptualization, Methodology, Software, Data curation, Formal analysis, Writing - original draft, Validation, Writing - review & editing.

## Declarations of competing interest

None.
